# Does adding immunotherapy to neoadjuvant chemotherapy increase postoperative morbidity in gastroesophageal junction adenocarcinoma? A propensity score-matched study

**DOI:** 10.3389/fimmu.2026.1806803

**Published:** 2026-07-03

**Authors:** Huiliang Zhang, Qingwen Huang, Jitao Du, Xiangbin Wan

**Affiliations:** Department of General Surgery, The Affiliated Cancer Hospital of Zhengzhou University & Henan Cancer Hospital, Zhengzhou, China

**Keywords:** gastroesophageal junction adenocarcinoma, immune-related adverse events, neoadjuvant immunotherapy, postoperative complications, propensity score matching, surgical safety

## Abstract

**Background:**

Landmark trials have established the surgical feasibility of neoadjuvant immunotherapy plus chemotherapy (NICT) in gastric and esophageal cancers, yet data specific to the narrow anatomical confines of the gastroesophageal junction (GEJ) remain limited. This study aimed to compare postoperative morbidity between patients receiving NICT and those receiving neoadjuvant chemotherapy (NCT) alone.

**Methods:**

This single-center, retrospective cohort study included consecutive patients with locally advanced GEJ adenocarcinoma (Siewert II/III) undergoing curative resection after NICT (n=140) or neoadjuvant chemotherapy alone (NCT, n=260). Propensity score matching (1:1) balanced baseline characteristics, including dMMR/MSI-H status and surgical approach, yielding 120 matched pairs. The primary outcome was major complications (Clavien-Dindo ≥III). Secondary outcomes included specific surgical complications, immune-related adverse events (irAEs), and interval to surgery.

**Results:**

After matching, NICT was associated with improved major pathological regression (58.3% vs. 45.8%; p=0.028). The primary endpoint was not significantly different between NICT and NCT cohorts (25.8% vs. 22.5%; p=0.538). Critically, no differences were observed in specific technical complications, including anastomotic leak (11.7% vs. 10.0%), conduit failure (3.3% vs. 2.5%), or pulmonary events. irAEs occurred in 13.3% of the NICT cohort without delaying surgical intervention. Multivariable and sensitivity analyses confirmed NICT was not an independent predictor of morbidity.

**Conclusion:**

This analysis provides anatomically focused evidence that the addition of immunotherapy to neoadjuvant chemotherapy for GEJ adenocarcinoma is not associated with a statistically significant increase in risk of major postoperative morbidity, anastomotic failure, or operative complexity. While larger randomized trials remain the gold standard, these findings support the extrapolation of established safety data to the technically demanding GEJ location.

## Introduction

The incidence of adenocarcinoma of the gastroesophageal junction (GEJ) has risen dramatically in recent decades, presenting a significant global health challenge (1). Despite advances in multimodal treatment, the prognosis for locally advanced Siewert type II and III adenocarcinomas remains suboptimal (2). The current standard of care of perioperative chemotherapy or neoadjuvant chemoradiotherapy has improved survival compared to surgery alone, yet recurrence rates remain high, and pathological complete response (pCR) rates with chemotherapy alone are often modest (3). Consequently, there is a continued need to optimize therapeutic strategies to enhance systemic control and downstage primary tumors prior to resection.

Immune checkpoint inhibitors (ICIs) have revolutionized the management of advanced esophageal and gastric cancers. Following their success in the metastatic and adjuvant settings, interest has rapidly shifted to the neoadjuvant setting, landmark studies, including the MATTERHORN, NEONIPIGA, and PANDA trials, have provided solid confirmatory evidence of surgical feasibility and improved pathological regression rates for upper gastrointestinal cancers (4–6). Theoretically, administering immunotherapy while the primary tumor and draining lymph nodes are intact may generate a stronger and more diverse anti-tumor immune response compared to adjuvant application (7).

However, the integration of immunotherapy into the neoadjuvant setting raises specific concerns for the surgical oncologist. The intense immune activation and subsequent tumor regression may induce distinct tissue alterations, such as dense fibrosis, hypervascularity, or tissue edema, potentially obliterating surgical planes and increasing the technical difficulty of dissection. Furthermore, immune-related adverse events (irAEs) affecting the lungs (pneumonitis) or endocrine system could theoretically compromise physiological reserve or delay the window for curative surgery (8–10).

While broad surgical feasibility has been established (4–6), data specifically addressing the surgical safety of NICT in the narrow anatomical confines of the GEJ remain limited. Because GEJ adenocarcinomas represent a unique entity at an anatomical and biological crossroads, extrapolating surgical and oncological outcomes from broader, mixed cohorts lacks the granularity required for precise surgical planning (11).

Therefore, the aim of this study was to provide granular, anatomically specific evidence evaluating the comparative surgical safety and short-term oncological outcomes of NICT versus neoadjuvant chemotherapy alone (NCT) specifically for locally advanced Siewert type II and III GEJ adenocarcinoma.

## Methods

### Study design and ethical oversight

This single-center, retrospective cohort study was conducted at Henan Cancer Hospital, a high-volume tertiary referral center for upper gastrointestinal malignancies. The study protocol was approved by the institutional review board, and informed written consent was obtained from all patients prior to initial treatment. The study was conducted in strict accordance with the Declaration of Helsinki and is reported following the Strengthening the Reporting of Observational Studies in Epidemiology guidelines.

### Study population

We identified consecutive adult patients (≥18 years) who underwent curative-intent surgical resection for adenocarcinoma of the GEJ between January 2017 and December 2023 from a prospectively maintained institutional surgical database. Eligible patients met the following inclusion criteria:

1. Histologically confirmed Siewert type II or III GEJ adenocarcinoma, with the tumor epicenter located within 5 cm (proximal or distal) of the anatomical cardia;
2. Clinical stage T2-4a, N0-3, M0 according to the AJCC 8th edition;
3. Completion of neoadjuvant systemic therapy followed by planned curative resection.

Exclusion criteria encompassed: Siewert type I tumors or pure esophageal/gastric body cancers; distant metastasis at diagnosis; emergency or palliative resection; prior major thoracic or upper abdominal surgery; or absence of requisite baseline/post-treatment staging data.

### Neoadjuvant treatment protocols

Patients were stratified into two cohorts based on the neoadjuvant regimen administered. Treatment allocation was determined by the institutional multidisciplinary tumor board based on current standards of care and patient eligibility.

In NICT Cohort, the specific ICIs administered were pembrolizumab (200 mg every 3 weeks), or sintilimab (200 mg every 3 weeks), or tislelizumab (200 mg every 3 weeks), penpulimab (200 mg every 3 weeks). Chemotherapy partners were selected at the discretion of the treating medical oncologist and consisted of either FOLFOX (infusional 5-fluorouracil, leucovorin, and oxaliplatin) every 2 weeks or CAPOX (capecitabine and oxaliplatin) every 3 weeks. All patients were scheduled to receive 3–4 cycles of neoadjuvant therapy prior to surgical resection. Concurrent radiotherapy (41.4–45 Gy) was integrated into the neoadjuvant regimen when two or more of the following criteria were present: (1) cT4a primary tumor with threatened circumferential resection margin on baseline endoscopic ultrasound; (2) bulky cN2-3 disease with radiographic evidence of extranodal extension; or (3) tumor length exceeding 5 cm on baseline imaging.

In NCT Cohort: Served as a contemporary control group receiving NCT alone (typically FLOT: 5-fluorouracil, leucovorin, oxaliplatin, and docetaxel). Patients with locally advanced disease requiring intensified local control received a CROSS-like regimen (carboplatin/paclitaxel with concurrent 41.4 Gy radiotherapy).

### Surgical resection and technique

All operations were performed or directly supervised by dedicated surgical oncologists. The surgical approach of transthoracic Ivor Lewis esophagectomy or transhiatal extended total gastrectomy was selected based on tumor location, surgeon expertise, and patient physiology. A standardized D2 lymph node dissection was performed in all cases. Restoration of continuity was achieved via a gastric conduit with a stapled intrathoracic or cervical esophagogastric anastomosis. A feeding jejunostomy tube was routinely placed for postoperative nutritional support.

### Data collection and outcome definitions

Clinical and pathological data were extracted from the electronic health record using a standardized, pre-piloted case report form. Baseline Variables included Age, sex, BMI, ASA physical status, smoking history, and clinical TNM stage (determined via EUS and CT/PET-CT). Pathological Outcomes included ypTNM stage, tumor regression grade (Mandard/Becker criteria), pCR, R0 resection status, and lymph node yield.

### Postoperative morbidity assessment

The primary outcome was postoperative complications occurring within 90 days of surgery. Complications were graded according to the Clavien-Dindo classification and defined per the ECCG benchmarks to ensure international comparability. Specific Complications included anastomotic leak (clinical signs confirmed by imaging/endoscopy), pulmonary events (pneumonia per CDC criteria, ARDS), cardiac events (arrhythmia requiring treatment, MI), chyle leak, and conduit failure. irAEs related to immunotherapy were adjudicated by a medical oncologist using CTCAE v5.0. Prior to each NICT cycle, patients underwent routine clinical and laboratory screening (including comprehensive metabolic panels, complete blood counts, and thyroid function tests) to detect subclinical toxicities. Additionally, preoperative restaging CT scans were reviewed by a multidisciplinary team for asymptomatic pneumonitis or colitis.

Secondary outcomes included length of postoperative stay, 90-day readmission, and 90-day mortality.

### Statistical analysis

Continuous variables are presented as mean ± SD or median (IQR) and compared using Student’s t-test or Mann-Whitney U test. Categorical variables are reported as frequencies (%) and compared using Chi-square or Fisher’s exact tests.

**Figure 1:**
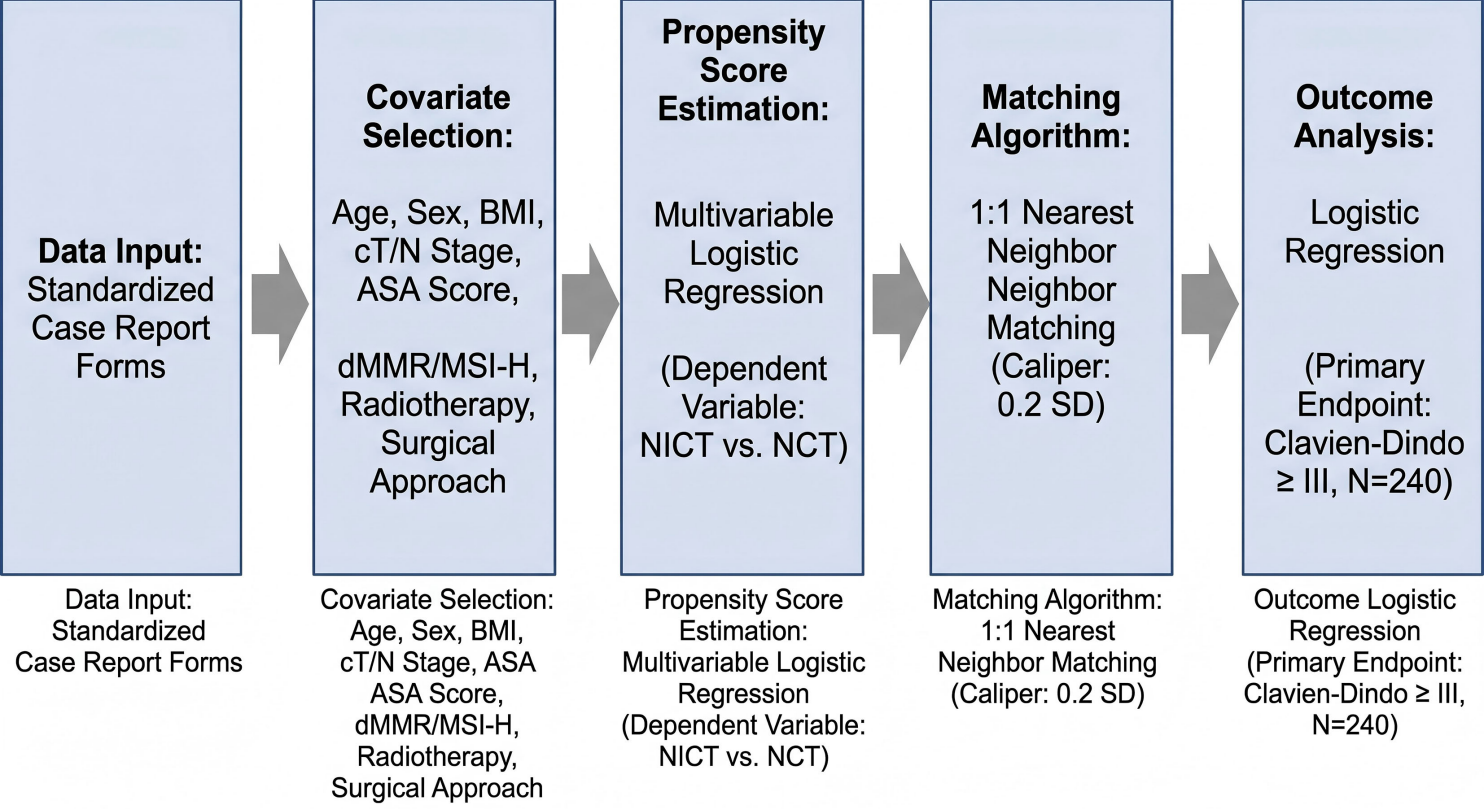
Analytical workflow pipeline. A schematic diagram illustrating the sequence of data intake, covariate selection, propensity score estimation, matching, and outcome analysis.

To mitigate selection bias inherent to the retrospective design, Propensity Score Matching (PSM) was performed. In propensity score estimation, a multivariable logistic regression model was used to estimate the probability of receiving NICT based on: age, sex, BMI, clinical T stage, clinical N stage, ASA score, dMMR/MSI-H, neoadjuvant radiotherapy usage, and surgical approach. A 1:1 nearest-neighbor matching algorithm was applied with a caliper width of 0.2 standard deviations of the logit of the propensity score. Covariate balance was assessed using standardized mean differences (SMD), with SMD < 0.1 indicating adequate balance. In the matched cohort, the primary outcome (Major Complications, Clavien-Dindo ≥ III) was compared using logistic regression, with results expressed as odds ratios (OR) and 95% confidence intervals (CI). Subgroup analyses for major postoperative complications were pre-specified based on clinically relevant baseline variables. Missing data were handled using complete case analysis. A two-sided p-value < 0.05 was considered statistically significant. Analyses were conducted using R software (v 4.5.2) and SPSS (v28.0) (**Figure 1**).

## Results

### Baseline data

**Figure 2:**
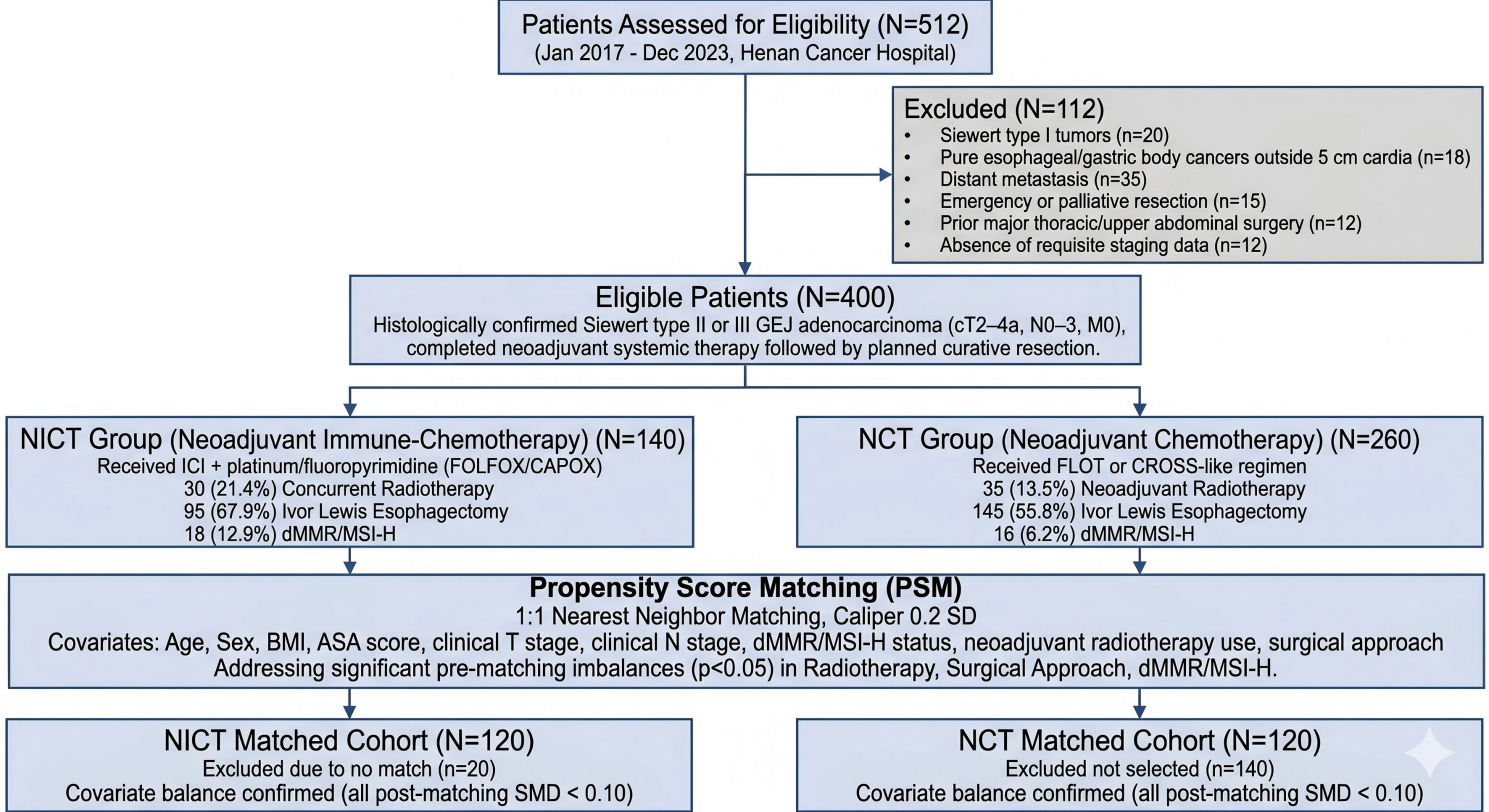
Patient selection flowchart. This figure details the screening process, inclusion/exclusion criteria, and the propensity score matching (PSM) algorithm used to create the matched NICT and NCT cohorts for analysis.

**Figure 3:**
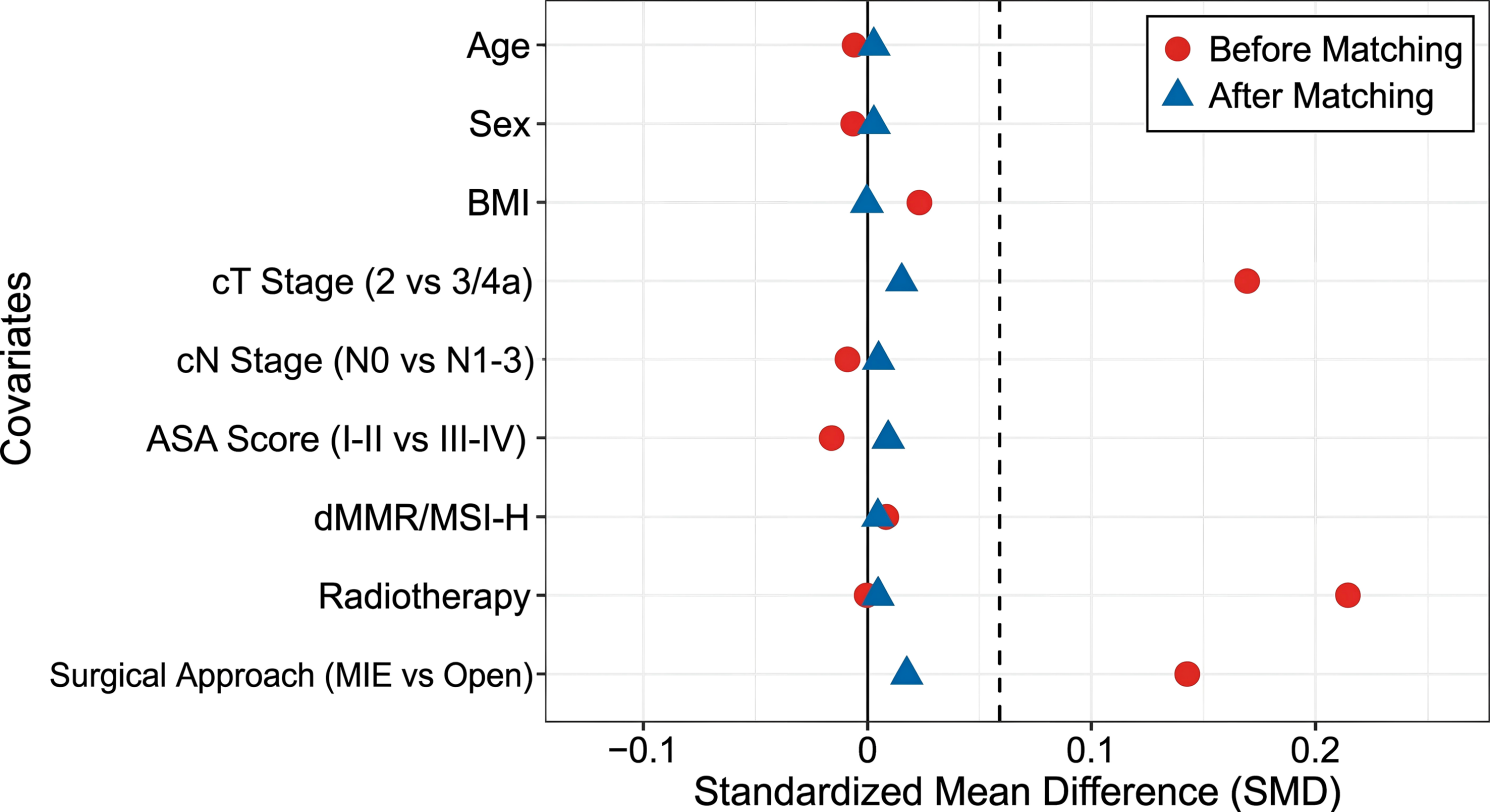
Love plot displaying standardized mean differences (SMD) for each covariate before (red circles) and after (blue triangles) propensity score matching. The vertical dashed line indicates an SMD of 0.1, a common threshold for assessing balance. All post-matching SMDs are <0.1, indicating excellent balance.

A total of 400 eligible patients were included in the study analysis, comprising 140 patients in the NICT cohort and 260 patients in the NCT cohort (**Figure 2**). Before matching, significant imbalances existed between the two groups regarding treatment modalities; patients in the NICT cohort were significantly more likely to receive neoadjuvant radiotherapy (21.4% vs. 13.5%; p=0.038) and undergo Ivor Lewis esophagectomy (67.9% vs. 55.8%; p=0.012) compared to those in the NCT cohort. The proportion of patients with dMMR/MSI-H status was also higher in the NICT cohort (12.9% vs. 6.2%, p=0.032). Other baseline variables, including age, sex, BMI, ASA score, and clinical TNM stage, were comparable between the groups (p > 0.05). After performing 1:1 PSM, 120 patients from each cohort were retained, resulting in a final matched population of 240 patients. The matching process successfully balanced all covariates, including dMMR/MSI-H status (10.0% vs. 8.3%, p=0.844). All post-matching SMD values were <0.10 with no statistically significant differences observed in the matched cohort for surgical approach (p=0.784) or neoadjuvant radiotherapy use (p=0.879) (**Table 1**; **Supplementary Table 1**; **Figure 3**).

**Table 1:** Baseline patient characteristics before and after propensity score matching.

Variable	Before Matching		After Matching		p-value (Before)	p-value (After)	SMD (After)
	NICT (n=140)	NCT (n=260)	NICT (n=120)	NCT (n=120)			
**Age, years**	63.2 ± 8.5	64.8 ± 9.0	63.5 ± 8.4	63.8 ± 8.7	0.074	0.782	0.035
**Sex, Male**	105 (75.0%)	200 (76.9%)	90 (75.0%)	89 (74.2%)	0.670	0.884	0.019
**BMI, kg/m²**	24.5 ± 3.6	25.0 ± 3.9	24.7 ± 3.5	24.6 ± 3.7	0.212	0.840	0.028
**ASA Score**					0.145	0.734	0.044
ASA I–II	115 (82.1%)	200 (76.9%)	100 (83.3%)	98 (81.7%)			
ASA III	25 (17.9%)	60 (23.1%)	20 (16.7%)	22 (18.3%)			
**Clinical T Stage**					0.328	0.864	0.022
cT2-3	115 (82.1%)	220 (84.6%)	100 (83.3%)	99 (82.5%)			
cT4a	25 (17.9%)	40 (15.4%)	20 (16.7%)	21 (17.5%)			
**Clinical N Stage**					0.415	0.877	0.017
cN0	60 (42.9%)	120 (46.2%)	52 (43.3%)	53 (44.2%)			
cN+	80 (57.1%)	140 (53.8%)	68 (56.7%)	67 (55.8%)			
**dMMR/MSI-H**	18 (12.9%)	16 (6.2%)	12 (10.0%)	10 (8.3%)	0.032	0.844	0.058
**Neoadjuvant Radiotherapy**	30 (21.4%)	35 (13.5%)	25 (20.8%)	24 (20.0%)	0.038	0.879	0.020
**Surgical Approach**					0.012	0.784	0.035
Ivor Lewis	95 (67.9%)	145 (55.8%)	82 (68.3%)	80 (66.7%)			
Transhiatal Gastrectomy	45 (32.1%)	115 (44.2%)	38 (31.7%)	40 (33.3%)			

NICT, Neoadjuvant Immunotherapy plus Chemotherapy; NCT, Neoadjuvant Chemotherapy; BMI, Body Mass Index; ASA, American Society of Anesthesiologists; dMMR/MSI-H, Mismatch Repair Deficient / Microsatellite Instability-High; SMD, Standardized Mean Difference.

Data are presented as mean ± standard deviation or n (%). P-values are derived from Student’s t-test or Mann-Whitney U test for continuous variables, and Chi-square or Fisher’s exact test for categorical variables, comparing the two cohorts before and after matching. SMD values are calculated for the matched cohorts; an SMD < 0.10 indicates adequate covariate balance.

### Pathological outcome

Although the distribution of ypT stages was statistically comparable between groups (p=0.621), the rate of ypT0 was numerically higher in the NICT cohort (23.3% vs. 16.7%). Similarly, nodal downstaging was evident, with 65.0% of patients in the NICT cohort achieving ypN0 status compared to 58.3% in the NCT cohort (p=0.187). Notably, the primary TRG significantly favored the NICT group (p=0.028); a higher proportion of patients in the NICT cohort achieved a complete or subtotal regression (TRG 1–2: 58.3%) compared to the NCT cohort (45.8%). The pCR rate was 21.7% in the NICT group versus 15.0% in the NCT group (p=0.178). R0 resection rates were high in both cohorts (95.8% vs. 94.2%; p=0.754), and the extent of lymphadenectomy was comparable, with a median lymph node yield of 30 (IQR 25–38) in the NICT group and 28 (IQR 23–35) in the NCT group (p=0.112) (**Table 2**).

**Table 2:** Pathological outcomes comparison.

Variable	NICT (n=120)	NCT (n=120)	P-value
ypT Stage			0.621
ypT0	28 (23.3%)	20 (16.7%)	
ypT1	38 (31.7%)	35 (29.2%)	
ypT2	25 (20.8%)	31 (25.8%)	
ypT3	24 (20.0%)	29 (24.2%)	
ypT4a	5 (4.2%)	5 (4.2%)	
ypN Stage			0.187
ypN0	78 (65.0%)	70 (58.3%)	
ypN1	26 (21.7%)	30 (25.0%)	
ypN2	12 (10.0%)	15 (12.5%)	
ypN3	4 (3.3%)	5 (4.2%)	
Tumor Regression Grade (Mandard/Becker)			**0.028**
TRG 1 (Complete regression)	28 (23.3%)	20 (16.7%)	
TRG 2 (Subtotal regression)	42 (35.0%)	35 (29.2%)	
TRG 3 (Partial regression)	36 (30.0%)	45 (37.5%)	
TRG 4 (Minimal regression)	12 (10.0%)	18 (15.0%)	
TRG 5 (No regression)	2 (1.7%)	2 (1.7%)	
Pathological Complete Response (pCR)	26 (21.7%)	18 (15.0%)	0.178
R0 Resection Rate	115 (95.8%)	113 (94.2%)	0.754
Lymph Node Yield, median (IQR)	30 (25-38)	28 (23-35)	0.112

NICT, Neoadjuvant Immunotherapy plus Chemotherapy; NCT, Neoadjuvant Chemotherapy; ypT, pathological T stage (post-neoadjuvant); ypN, pathological N stage (post-neoadjuvant); TRG, Tumor Regression Grade; pCR, pathological Complete Response; R0, microscopically negative resection margin; IQR, Interquartile Range. Bold value means p<0.05.

### Postoperative morbidity and safety profiles

**Figure 4:**
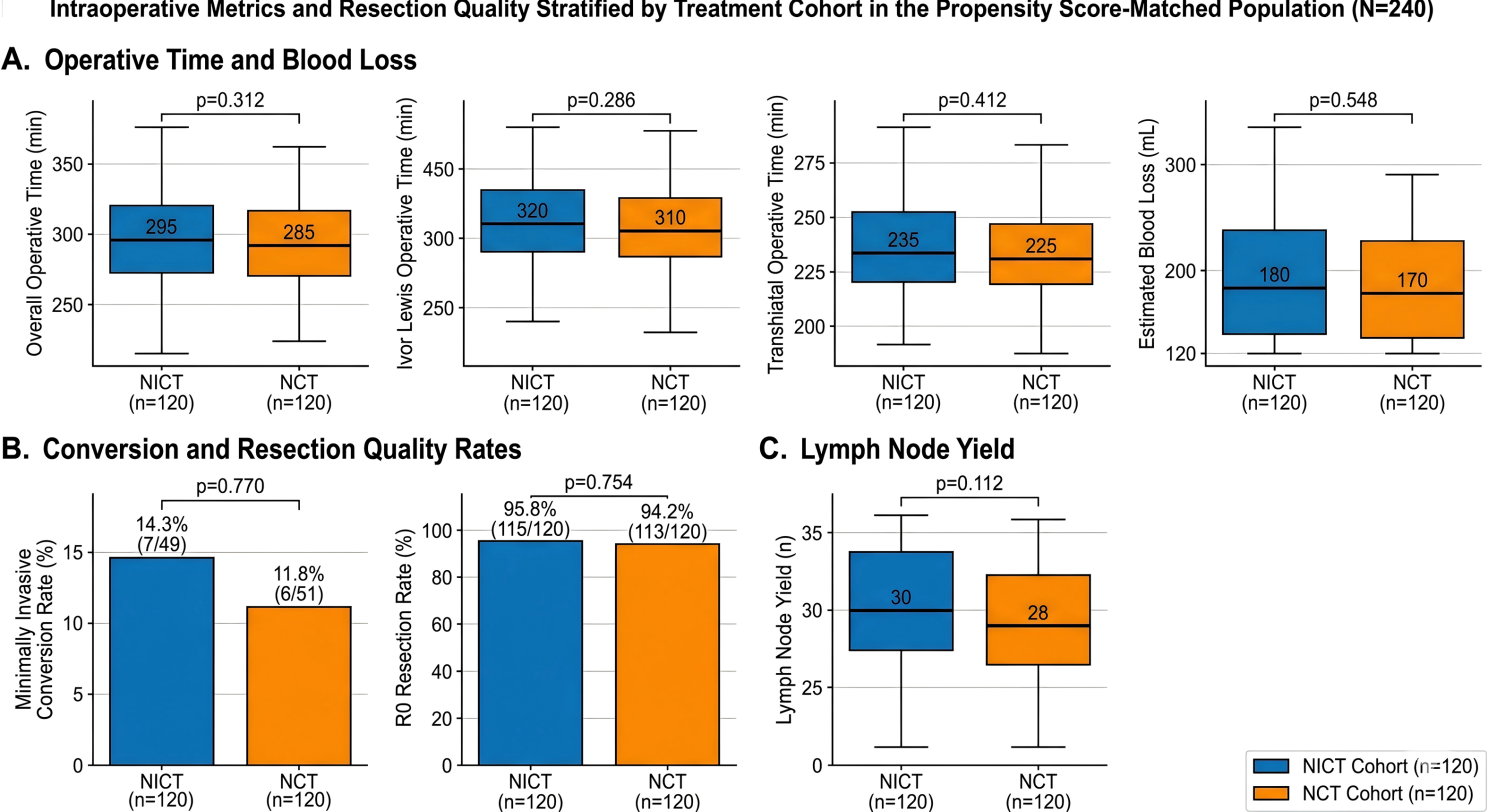
Intraoperative metrics and resection quality stratified by treatment cohort in the propensity score-matched population (N = 240). **(A)** for operative time and blood loss; **(B)** for conversion and resection quality rates; **(C)** for lymph node yield.

Median operative time was 295 minutes (IQR 255--340) in the NICT group versus 285 minutes (IQR 250--330) in the NCT group (p=0.312). When stratified by surgical approach, operative times for Ivor Lewis esophagectomy (median 320 vs. 310 minutes; p=0.286) and transhiatal gastrectomy (median 235 vs. 225 minutes; p=0.412) were similar. Median estimated blood loss was 180 mL (IQR 120--280) with NICT compared to 170 mL (IQR 120--250) with NCT (p=0.548). Among patients undergoing a minimally invasive approach, conversion to open surgery occurred in 7 of 49 patients (14.3%) in the NICT group and 6 of 51 patients (11.8%) in the NCT group (p=0.770) (**Figure 4**).

**Figure 5:**
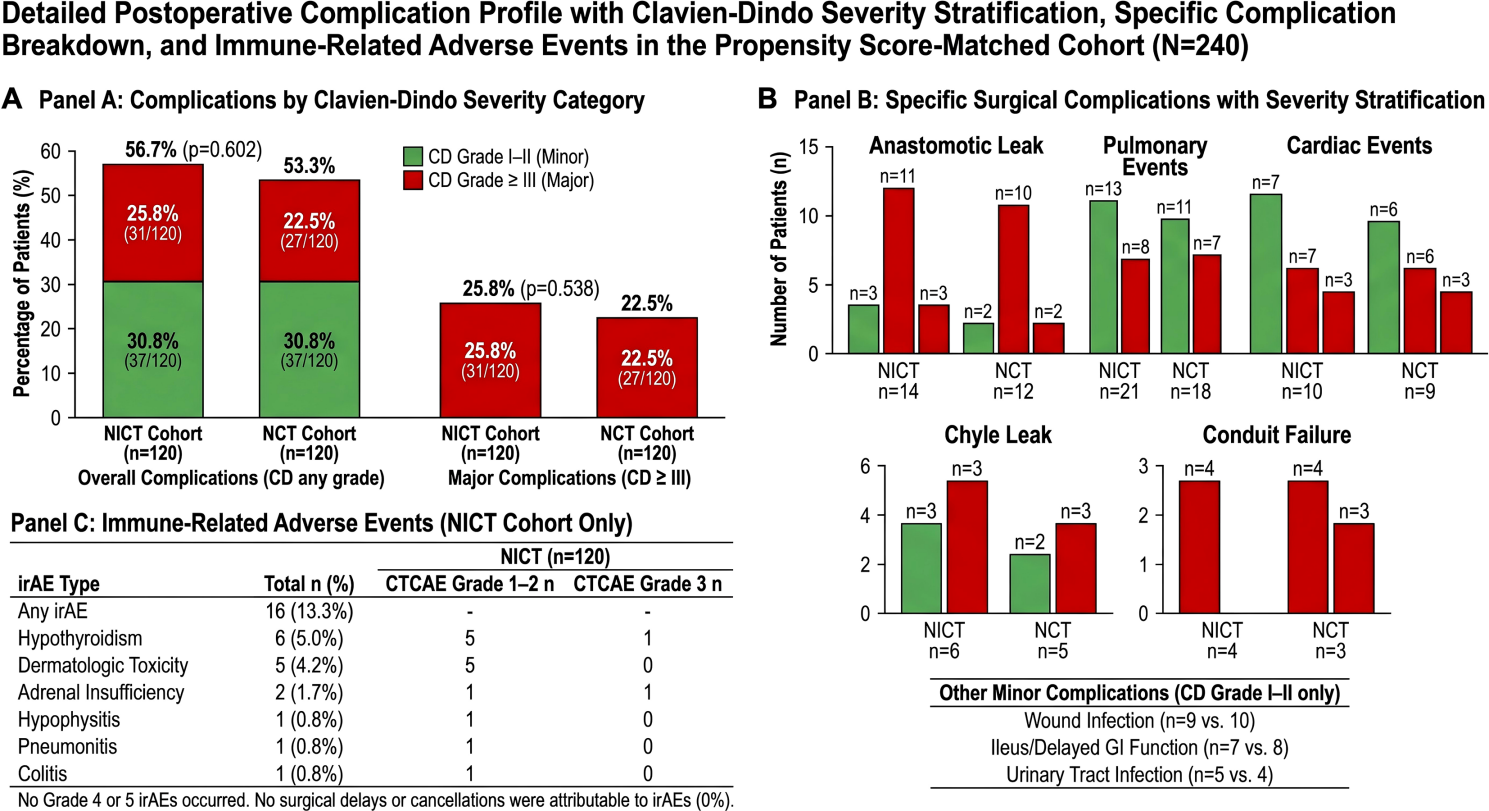
Detailed postoperative complication profile with clavien-dindo severity stratification, specific complication breakdown, and immune-related adverse events in the propensity score-matched cohort (N = 240). **(A)** for complication; **(B)** for specific complication; **(C)** for immuno-related adverse events.

The overall incidence of postoperative complications was comparable between the two cohorts (NICT: 56.7% vs. NCT: 53.3%; p=0.602). Similarly, there was no statistically significant difference in the rate of major complications (Clavien-Dindo ≥ III) between the NICT (25.8%) and NCT (22.5%) groups (p=0.538). Specific surgical complications, including anastomotic leakage (11.7% vs. 10.0%; p=0.678), pulmonary events (17.5% vs. 15.0%; p=0.596), and conduit failure (3.3% vs. 2.5%; p=0.702), showed no significant variance attributable to the addition of immunotherapy. In the NICT cohort, 16 (13.3%) patients experienced at least one immune-related adverse event. There were no surgical delays or cancellations. Hypothyroidism was the most frequently observed irAE, occurring in 6 patients (5.0%), of which five cases were Grade 1–2 and one case was Grade 3. Dermatologic toxicity manifesting as rash or pruritus occurred in 5 patients (4.2%), all of which were Grade 1–2. Adrenal insufficiency was documented in 2 patients (1.7%), with one Grade 1–2 and one Grade 3 event. Single cases of Grade 1–2 hypophysitis, pneumonitis, and colitis were each observed in 1 patient (0.8%) (**Figure 5**).

Secondary outcomes, including median length of stay (13 vs. 12 days; p=0.285) and 90-day mortality (4.2% vs. 3.3%; p=0.734), were similar between groups (**Table 3**).

**Table 3:** Postoperative morbidity and complications after propensity score Matching.

Variable	NICT (n=120)	NCT (n=120)	P-value
Overall Complication Rate (Clavien-Dindo Any Grade)	68 (56.7%)	64 (53.3%)	0.602
Major Complications (Clavien-Dindo ≥ Grade III)	31 (25.8%)	27 (22.5%)	0.538
Specific Complications			
Anastomotic Leak	14 (11.7%)	12 (10.0%)	0.678
Pulmonary Events (Pneumonia, ARDS)	21 (17.5%)	18 (15.0%)	0.596
Cardiac Events (Arrhythmia, MI)	10 (8.3%)	9 (7.5%)	0.815
Chyle Leak	6 (5.0%)	5 (4.2%)	0.762
Conduit Failure	4 (3.3%)	3 (2.5%)	0.702
irAEs – NICT Group Only	16 (13.3%)	–	–
Secondary Outcomes			
Length of Postoperative Stay, median (IQR), days	13 (10–19)	12 (9–18)	0.285
90-Day Readmission Rate	17 (14.2%)	14 (11.7%)	0.558
90-Day Mortality	5 (4.2%)	4 (3.3%)	0.734

NICT, Neoadjuvant Immunotherapy plus Chemotherapy; NCT, Neoadjuvant Chemotherapy; ARDS, Acute Respiratory Distress Syndrome; MI, Myocardial Infarction; irAEs, Immune-Related Adverse Events; IQR, Interquartile Range.

### Predictors of major postoperative morbidity

In univariable analysis, no baseline demographic or clinicopathological variable—including age, ASA physical status, clinical stage, or MMR/MSI status—demonstrated a statistically significant association with severe morbidity. To strictly control for residual confounding, a multivariable model was constructed adjusting for age, sex, BMI, ASA score, clinical TNM stage, neoadjuvant radiotherapy, and surgical approach. Consistent with the unadjusted findings, NICT was not independently associated with an increased risk of major complications compared to NCT (adjusted odds ratio [aOR] 1.18; 95% CI 0.64–2.21; p=0.589). Furthermore, neither neoadjuvant radiotherapy (aOR 1.22; p=0.599) nor surgical approach (aOR 1.01; p=0.973) emerged as significant predictors of morbidity. The regression model demonstrated good calibration (Hosmer-Lemeshow test, p=0.892) but critically low discriminatory power (Nagelkerke R² = 0.021; AUC = 0.542), indicating that major complications in this matched cohort were not predicted by the neoadjuvant regimen or measured baseline characteristics, and were likely dominated by unmeasured perioperative and technical variables (**Table 4**).

**Table 4:** Univariate and multivariable analyses for major complications (Clavien-Dindo ≥ III) in the matched cohort (N=240).

Variable	Category / Unit	Univariate		Multivariable	
		OR (95% CI)	p-value	aOR (95% CI)	P-value
Treatment group					
	NCT (Ref)	1.00	–	1.00	–
	NICT	1.20 (0.66–2.18)	0.538	1.18 (0.64–2.21)	0.589
Age	per 1-year increase	1.01 (0.98–1.04)	0.694	1.01 (0.97–1.04)	0.722
Sex					
	Female (Ref)	1.00	–	1.00	–
	Male	0.97 (0.50–1.88)	0.925	0.96 (0.48–1.91)	0.906
BMI	per 1 kg/m² increase	1.02 (0.94–1.10)	0.612	1.02 (0.94–1.11)	0.592
ASA score					
	ASA I–II (Ref)	1.00	–	1.00	–
	ASA III	1.32 (0.63–2.78)	0.464	1.30 (0.61–2.77)	0.498
Clinical T stage					
	cT2–3 (Ref)	1.00	–	1.00	–
	cT4a	0.86 (0.39–1.91)	0.715	0.88 (0.39–2.01)	0.765
Clinical N stage					
	cN0 (Ref)	1.00	–	1.00	–
	cN+	0.94 (0.52–1.71)	0.846	0.93 (0.50–1.73)	0.819
**MMR/MSI status**	MSS/MSI-L (Ref)	1.00		1.00	
	dMMR/MSI-H	0.85 (0.32-2.26)	0.742	0.82 (0.30-2.23)	0.698
Neoadjuvant radiotherapy					
	No (Ref)	1.00	–	1.00	–
	Yes	1.17 (0.57–2.38)	0.670	1.22 (0.59–2.53)	0.599
Surgical approach					
	Transhiatal Gastrectomy (Ref)	1.00	–	1.00	–
	Ivor Lewis Esophagectomy	0.98 (0.53–1.83)	0.956	1.01 (0.53–1.92)	0.973

NICT, neoadjuvant immunochemotherapy; NCT, neoadjuvant chemotherapy; OR, Odds Ratio; aOR, Adjusted Odds Ratio; CI, Confidence Interval; Ref, Reference category; dMMR/MSI-H, Mismatch Repair Deficient / Microsatellite Instability-High; MSS/MSI-L, Microsatellite Stable / Microsatellite Instability-Low. Model Performance for Multivariable Analysis, Hosmer–Lemeshow test: p=0.892 (good fit). Nagelkerke R^2^: 0.021. C-statistic (AUC): 0.542.

### Subgroup analysis

**Figure 6:**
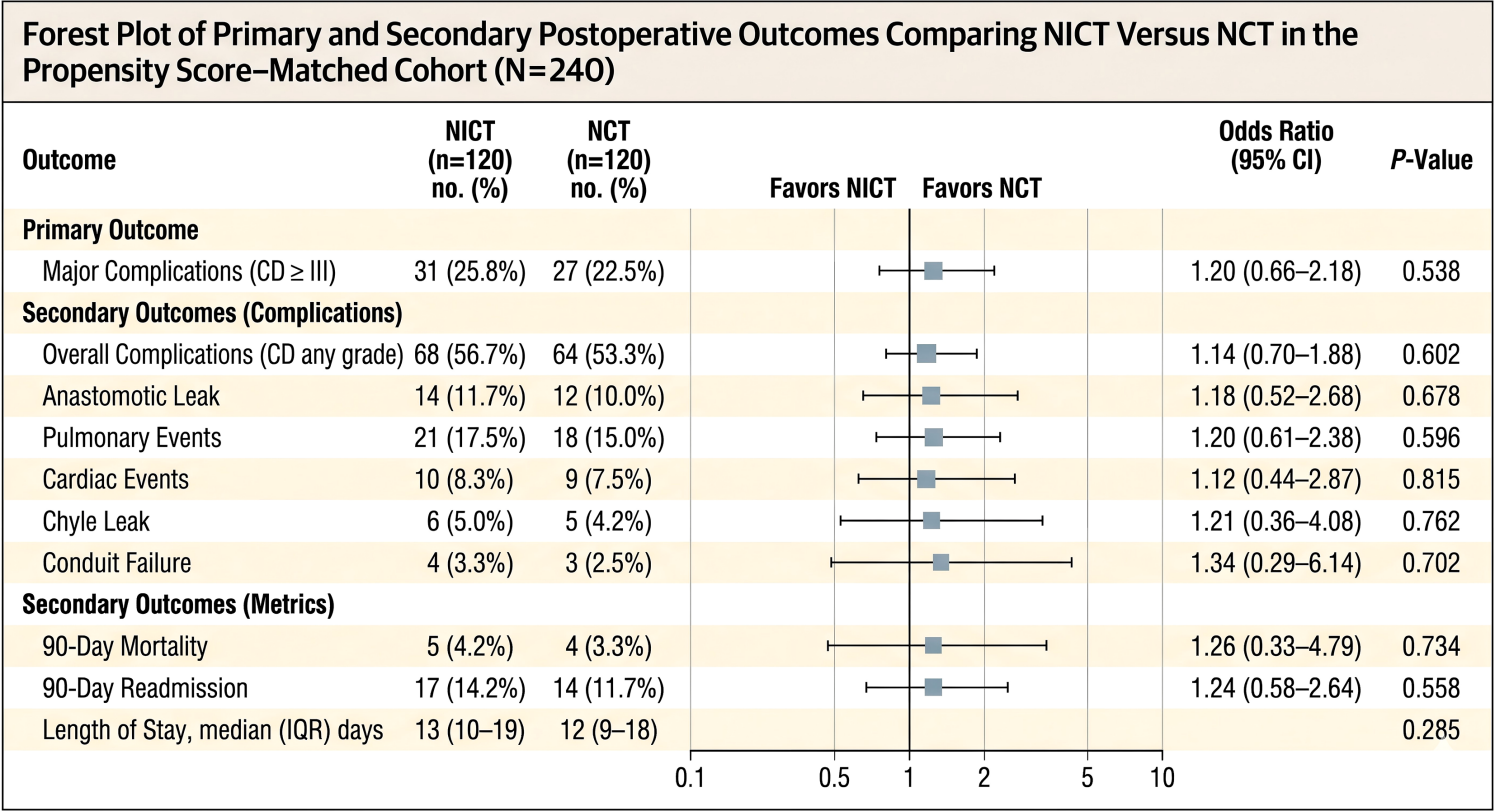
Forest plot of primary and secondary postoperative outcomes comparing NICT versus NCT in the propensity score-matched cohort (N = 240).

The stratified analysis revealed consistent findings across various clinically relevant subgroups. The risk of major postoperative complications was not significantly different between the NICT and NCT cohorts, regardless of the surgical approach (Ivor Lewis esophagectomy vs. transhiatal gastrectomy), baseline clinical T or N stage, use of neoadjuvant radiotherapy, dMMR/MSI-H status, or the degree of pathological tumor response (TRG 1-2 vs. 3-5). Adjusted odds ratios within each stratum closely approximated the primary estimate from the overall matched cohort (aOR 1.18), with all confidence intervals crossing unity and p-values for interaction exceeding 0.05 (**Table 5**; **Figure 6**).

**Table 5:** Subgroup analysis for major complications (Clavien-Dindo ≥ III).

Subgroup	NICT (n/N)	NCT (n/N)	Adjusted OR (95% CI)	P-value for Interaction
Surgical approach				
Ivor Lewis Esophagectomy	20/82	18/80	1.15 (0.55–2.39)	0.836
Transhiatal Gastrectomy	11/38	9/40	1.24 (0.46–3.32)	
Clinical T stage				
cT2–3	24/100	22/99	1.12 (0.58–2.17)	0.724
cT4a	7/20	5/21	1.45 (0.38–5.53)	
Clinical N stage				
cN0	12/52	11/53	1.10 (0.43–2.77)	0.804
cN+	19/68	16/67	1.23 (0.57–2.65)	
Neoadjuvant radiotherapy				
Yes	7/25	6/24	1.20 (0.33–4.36)	0.953
No	24/95	21/96	1.17 (0.60–2.29)	
TRG				
TRG 1–2 (Major Response)	16/70	13/55	1.24 (0.54–2.84)	0.889
TRG 3–5 (Minor/No Response)	15/50	14/65	1.19 (0.51–2.79)	
MMR/MSI status				
dMMR/MSI-H	3/12	2/10	1.25 (0.18-8.61)	0.927
MMR/MSI-L	28/108	25/110	1.17 (0.63-2.19)	

All subgroup analyses were adjusted for age, sex, BMI, ASA score, and clinical TNM stage (except for the variable being stratified). Odds Ratios (OR) compare NICT vs. NCT within each stratum. TRG: Tumor Regression Grade. dMMR/MSI-H: Mismatch Repair Deficient / Microsatellite Instability-High; MSS/MSI-L: Microsatellite Stable / Microsatellite Instability-Low.

### Sensitivity analysis

Sensitivity analyses, including excluding patients with preoperative irAEs, applying alternative propensity score matching calipers, handling missing data via multiple imputation, using inverse probability of treatment weighting, restricting the analysis to patients not receiving radiotherapy, employing a stricter definition of major complications, and multivariable logistic regression on the entire pre-matching cohort all yielded results congruent with the main analysis. The adjusted odds ratios for major complications associated with NICT remained statistically non-significant across all scenarios, with point estimates consistently close to 1.0 and confidence intervals consistently encompassing the null value (**Table 6**).

**Table 6:** Sensitivity analyses for primary outcome (major complications).

Sensitivity analysis	Cohort / Method	NICT (n/N)	NCT (n/N)	Adjusted OR (95% CI)	P
1. Excluding Patients with Pre-op irAEs	Matched cohort, excluding NICT irAEs (n=104)	27/104	27/120	1.15 (0.61–2.17)	0.664
2. PSM with Different Calipers					
Caliper = 0.1 SD	Re-matched cohort (n=210)	26/105	22/105	1.23 (0.64–2.37)	0.531
Caliper = 0.3 SD	Re-matched cohort (n=250)	32/125	28/125	1.18 (0.66–2.13)	0.576
3. Multiple Imputation for Missing Data	Full cohort (N=400), MICE	31/140	27/260	1.14 (0.70–1.86)	0.601
4. Inverse Probability of Treatment Weighting (IPTW)	Full cohort (N=400), IPTW	–	–	1.09 (0.73–1.63)	0.681
5. Excluding Patients with Neoadjuvant Radiotherapy	Matched cohort, no RT (n=191)	24/95	21/96	1.17 (0.60–2.29)	0.641
6. Major Complications as CD ≥ IV	Matched cohort (n=240)	12/120	10/120	1.22 (0.50–2.97)	0.665
7.Multivariable Regression on Full Pre-PSM Cohort	Full cohort (N=400), Adjusted Model	34/140	60/260	1.41 (0.71-1.85)	0.589

NICT, Neoadjuvant Immunotherapy plus Chemotherapy; NCT, Neoadjuvant Chemotherapy; OR, Odds Ratio; CI, Confidence Interval; PSM, Propensity Score Matching; SD, Standard Deviation; MICE, Multiple Imputation by Chained Equations; IPTW, Inverse Probability of Treatment Weighting; irAEs, Immune-Related Adverse Events; RT, Radiotherapy; CD, Clavien-Dindo. For analyses using the full cohort (IPTW, MICE), model adjustments included age, sex, BMI, ASA score, clinical TNM stage, neoadjuvant radiotherapy, and surgical approach.

### Immunological biomarker analysis

To identify which patients derived the greatest pathological benefit from NICT, we analyzed baseline PD-L1 Combined Positive Score (CPS) and HER2 status for all 140 patients in the NICT cohort. The median PD-L1 CPS was 8.0 (IQR 2.0–18.0). A clinically meaningful gradient of response was observed across CPS subgroups, with the highest rates of major pathological response (TRG 1-2) and pCR concentrated in patients with higher PD-L1 expression (**Table 7**). Specifically, patients with a CPS ≥5 (n=81, 57.9%) achieved a major pathological response rate of 66.7% (54/81) compared to 35.6% (21/59) in the CPS <5 group (p<0.001). The pCR rate was 27.2% (22/81) versus 6.8% (4/59), respectively (p=0.002). Conversely, HER2-positive tumors (n=18, 12.9%) demonstrated a pattern of profound resistance to NICT. No patient with HER2-positive disease achieved a pCR (0/18 vs. 26/122 [21.3%] in HER2-negative; p=0.022), and the major pathological response rate was only 16.7% (3/18) compared to 59.0% (72/122) in the HER2-negative subgroup (p<0.001). Combining biomarkers, the CPS ≥5/HER2-negative subgroup (n=75, 53.6%) achieved a 69.3% major pathological response rate and a 29.3% pCR rate, whereas the small CPS <5/HER2-positive subgroup (n=7, 5.0%) had a 0% response rate. Importantly, the rate of major postoperative complications (Clavien-Dindo ≥ III) did not differ significantly across any biomarker-defined subgroup within the full NICT cohort (CPS <5: 23.7% vs. CPS ≥5: 21.0%, p=0.635; HER2-positive: 27.8% vs. HER2-negative: 21.3%, p=0.829).

**Table 7:** Pathological response and postoperative morbidity in the full NICT cohort stratified by baseline PD-L1 CPS and HER2 status (n=140).

Variable	n (%)	TRG 1-2 (Major Response)	pCR	Major complications (CD ≥ III)
Overall NICT Cohort	140 (100)	75 (53.6)	26 (18.6)	31 (22.1)
PD-L1 CPS Category		p < 0.001	p = 0.002	p = 0.635
CPS < 1	28 (20.0)	11 (39.3)	2 (7.1)	7 (25.0)
CPS 1–4	31 (22.1)	10 (32.3)	2 (6.5)	7 (22.6)
CPS 5–9	32 (22.9)	20 (62.5)	7 (21.9)	6 (18.8)
CPS ≥ 10	49 (35.0)	34 (69.4)	15 (30.6)	11 (22.4)
Dichotomized CPS		p < 0.001	p = 0.002	p = 0.704
CPS < 5	59 (42.1)	21 (35.6)	4 (6.8)	14 (23.7)
CPS ≥ 5	81 (57.9)	54 (66.7)	22 (27.2)	17 (21.0)
HER2 Status		p < 0.001	p = 0.022	p = 0.829
HER2-Positive	18 (12.9)	3 (16.7)	0 (0.0)	5 (27.8)
HER2-Negative	122 (87.1)	72 (59.0)	26 (21.3)	26 (21.3)
Combined Profiles		p < 0.001	p < 0.001	p = 0.758
CPS ≥ 5 & HER2-Negative	75 (53.6)	52 (69.3)	22 (29.3)	16 (21.3)
CPS < 5 & HER2-Positive	7 (5.0)	0 (0.0)	0 (0.0)	2 (28.6)

NICT, Neoadjuvant Immunotherapy plus Chemotherapy; CPS, Combined Positive Score; TRG, Tumor Regression Grade; pCR, pathological Complete Response; HER2, Human Epidermal growth factor Receptor 2; CD, Clavien-Dindo.

P-values for trend across ordinal CPS categories (Cochran-Armitage test) reported in the first row; all other p-values from Chi-square or Fisher's exact test.

## Discussion

In this PSM analysis of locally advanced GEJ adenocarcinoma, we evaluated the surgical safety and short-term oncological efficacy of NICT. While recent landmark trials have established the general surgical feasibility of neoadjuvant immunotherapy without raising alarming safety signals (4–6), our study adds much-needed granularity in a specific, high-risk anatomical context. By positioning surgical safety as the primary question rather than an afterthought, we evaluated a breadth of specific endpoints beyond global Clavien-Dindo grades, including anastomotic healing, conduit failure, and irAE-to-surgery intervals. Within this robust framework, we demonstrated that NICT yielded a significantly higher rate of major pathological regression (TRG 1-2: 58.3% vs. 45.8%; p=0.028) compared to NCT, without a statistically significant increase in major postoperative complications (25.8% vs. 22.5%; p=0.538). Furthermore, despite theoretical concerns regarding immunotherapy-induced fibrosis and irAEs, we observed no increase in anastomotic leakage, conduit failure, or perioperative mortality.

The primary concern regarding NICT has been whether the systemic immune activation translates into local tissue toxicity, thereby impairing anastomotic healing or increasing susceptibility to infection. Our major complication rate of 25.8% in the NICT group is a critical data point that requires contextualization within the global landscape. When compared to large-scale international benchmarks, our safety data is highly favorable. A study of the Esophageal Complications Consensus Group involving over 2,700 esophagectomies across 24 countries (12), reported an overall major morbidity rate ranging between 29% and 35% for standard neoadjuvant therapies. Our rate of 25.8% sits at the lower end of this spectrum, suggesting that NICT does not deviate from historical safety standards established by chemotherapy or chemoradiotherapy. Similarly, Stiles et al. (13) reported complication rates exceeding 45% in Western cohorts undergoing trimodality therapy, further reinforcing that our outcomes achieved in a high-volume Asian center demonstrate that NICT is not inherently more dangerous than established standards of care. While our complication rates are reassuring, they must be interpreted in the context of statistical power. The adjusted odds ratio of 1.18 for major morbidity carried a wide 95% confidence interval (0.64–2.21), encompassing up to a doubling of risk. With 120 patients per group and a baseline major complication rate of 23%, this study had 80% power to detect an absolute risk increase of approximately 16 percentage points at a two-sided alpha of 0.05. Smaller, yet clinically meaningful differences would require over 350 patients per arm to reliably exclude. The non-significant result, therefore, reflects insufficient power rather than demonstrated safety, and equivalence between regimens cannot be inferred from these data.

Specific attention must be paid to anastomotic leakage, the “Achilles’ heel” of GEJ surgery. Our leak rate (11.7% in NICT vs. 10.0% in NCT) showed no significant difference. This aligns with recent findings from Li et al. (14) and Zhang et al. (15), who investigated NICT in Chinese cohorts and reported leak rates of 8.9% and 12.3% respectively, concluding that PD-1 blockade does not impair microvascular perfusion or collagen deposition at the anastomosis. Conversely, Plum et al. (16) highlighted that in Western populations, leak rates can be influenced heavily by comorbidities such as BMI and diabetes; the fact that our matched cohort balanced these factors strengthens the conclusion that the drug regimen itself is not the driver of anastomotic failure.

A qualitative concern frequently cited by surgeons is the phenomenon of “hostile” surgical planes. The robust immune response induced by PD-1 inhibitors can theoretically lead to dense hypervascular fibrosis, particularly around the hilum and metastatic lymph nodes, potentially obliterating dissection planes. Mays et al. (17) described this phenomenon in a series of lung cancer resections following immunotherapy, noting that “dense hilar fibrosis” significantly increased the technical difficulty of vascular dissection and the risk of conversion to open surgery. Similarly, Larouche et al. (18) reported that in a subset of esophageal patients, post-immunotherapy tissues exhibited increased friability and neovascularization, which could theoretically prolong operative times and increase blood loss. However, our quantitative data indirectly refutes the notion that this fibrosis compromises surgical safety in GEJ adenocarcinoma. We observed no significant differences in operative time, estimated blood loss, or R0 resection rates between the NICT and NCT groups. This discrepancy between the “fear” of fibrosis and the “reality” of surgical outcomes is supported by Barjot et al. (19), who evaluated the feasibility of surgery after immunotherapy. They concluded that while tissue texture changes are common, they rarely render the tumor unresectable or lead to catastrophic intraoperative adverse events when performed by experienced surgical teams. The discrepancy between theoretical concerns of “hostile” surgical planes and our favorable clinical outcomes likely stems from anatomical differences between GEJ adenocarcinomas and more proximal thoracic malignancies. Resections for ESCC frequently involve the mid-to-upper mediastinum, where immunotherapy-induced hypervascular fibrosis and reactive lymphadenopathy can severely tether the esophagus to critical structures like the membranous trachea or aortic arch. In contrast, GEJ surgery primarily targets the lower mediastinum and upper abdomen. Fascial planes surrounding the distal esophagus and gastric cardia are mechanistically less susceptible to the dense fibrosis observed in pulmonary hilar or upper mediastinal dissections (17). Alternatively, the specific chemotherapy backbone may attenuate the local inflammatory response.

Another unique risk of NICT is the potential for irAEs to delay or cancel curative surgery. Fuentes et al. (20) emphasized that severe irAEs, such as pneumonitis, hepatitis, or hypophysitis, can occur unpredictably and may require high-dose corticosteroids, thereby pushing the patient out of the optimal surgical window or increasing the risk of postoperative infection. Yip et al. (21) similarly cautioned that even low-grade irAEs could complicate perioperative management, particularly regarding adrenal sufficiency. In our study, 13.3% of NICT patients experienced irAEs, yet notably, 0% experienced a delay in surgery. This favorable outcome likely reflects the relatively short duration of neoadjuvant exposure (typically 3–4 cycles) used in our protocol, which may be the “sweet spot” for efficacy without toxicity. Chen et al. (22) proposed the concept of a “surgical window of opportunity” for immunotherapy, suggesting that limiting neoadjuvant cycles minimizes the cumulative risk of high-grade irAEs while still priming the T-cell response. Our data supports this strategy. Furthermore, strict preoperative screening for endocrinopathies, as suggested by Yip et al. (21), allowed us to manage mild thyroid dysfunction medically without postponing resection.

Pathological Response as a Surrogate While safety was the primary endpoint, the driving force for adopting NICT is oncological efficacy. We observed a pCR rate of 21.7% in the NICT group compared to 15.0% in the NCT group. While this numerical increase did not reach statistical significance (p=0.178) likely due to sample size, the significant improvement in Major Pathological Response (TRG 1-2: 58.3% vs 45.8%) indicates a profound biological effect. These results are consistent with the broader literature but also highlight heterogeneity in response. Chen et al. (23) and Lei et al. (24) performed meta-analyses confirming that neoadjuvant immunotherapy significantly increases pCR rates across esophageal and gastric cancers. However, some phase II trials (25) have reported pCR rates approaching 30–35%. Contextualizing our pathological outcomes requires acknowledging the biological dichotomy between GEJ adenocarcinomas and highly immunogenic ESCC, which frequently achieve pCR rates exceeding 30–40% following NICT. Our cohort’s slightly lower pCR rate (21.7%) likely reflects the inclusion of Siewert type III tumors. These malignancies biologically mirror gastric cancers characterized by a heterogeneous, immunosuppressive microenvironment and distinct spatial profiles of HER2 and PD-L1 expression making them notoriously less responsive to systemic therapy. Therefore, achieving a 21.7% pCR rate should not be viewed as modest when compared to ESCC trials; rather, it signifies a profound biological shift for a tumor type historically recalcitrant to systemic downstaging. The clinical relevance of these pathological findings is underscored by Takada et al. (26), who demonstrated a linear correlation between pathological regression grade and long-term overall survival in GEJ adenocarcinoma. By shifting a significant proportion of patients from TRG 3–5 to TRG 1–2, NICT enhances early local tumor regression, though long-term oncological outcomes and survival benefits require further validation. The underlying mechanism, as detailed by Raziyeva et al. (27), involves the reactivation of exhausted CD8+ T-cells within the tumor microenvironment, creating a systemic memory response that may control micrometastatic disease better than chemotherapy alone.

The heterogeneity in our pathological response rates points to the urgent need for better patient selection. Not all patients benefit from NICT, yet all are exposed to its risks. Greene et al. (28) and Lacina et al. (29) have argued that PD-L1 expression and MSI status should be mandatory stratification factors. In our study, we matched for dMMR/MSI-H status, ensuring that the safety comparison was not skewed by the hyper-responsive MSI-H population. However, we did not stratify by CPS score. In fact, HER2 and PD-L1 expression profiles in GEJ cancers are distinct from gastric body cancers, suggesting that future trials must treat GEJ as a unique biological entity rather than lumping it with gastric or esophageal cohorts. The varying efficacy reported in Li et al. (30) versus our study may well be driven by unmeasured differences in PD-L1 prevalence between cohorts.

PD-L1 CPS and HER2 status are critical for understanding spatial tumor profiles and stratifying immune responses in upper gastrointestinal malignancies (1, 28, 29). Consistent with the distinct immunobiological profiles of GEJ adenocarcinomas, tumor regression was heavily dependent on these baseline biomarkers. Tumors with PD-L1 CPS ≥5 demonstrated significantly higher major pathological response and pCR rates compared to the CPS <5 subgroup. Conversely, HER2-positivity conferred profound resistance to immunotherapy, yielding no pathological complete responses. Integrating these profiles isolated a highly responsive CPS ≥5/HER2-negative cohort and a uniformly resistant CPS <5/HER2-positive subset. Crucially, despite marked variations in localized immune activation and subsequent tissue regression which were theoretically associated with dense fibrosis and increased surgical complexity (17, 18), the incidence of major postoperative morbidity remained statistically uniform across all biomarker strata. These findings indicate that while the immunobiological microenvironment strictly dictates the oncological efficacy of NICT, the localized biological cost of this immune response does not subsequently compromise surgical safety (14, 15).

Several limitations must be acknowledged. First, despite the robustness of PSM, this remains a retrospective single-center study. Selection bias regarding performance status or surgeon preference for specific approaches cannot be fully eliminated. Crucially, the poor discriminatory power of our multivariable model provides strong statistical evidence that the primary outcome was driven by unmeasured or inherently unpredictable factors absent from our dataset. Key intraoperative variables known to influence major morbidity, such as specific anastomotic technique (hand-sewn vs. stapled), individual surgeon volume, precise operative time, and estimated blood loss, were not included in the primary model. Their absence likely explains the model’s failure to outperform a coin flip, and this represents a significant analytical limitation. Second, our comparison with Western literature requires caution due to differences in patient BMI, genetic backgrounds, and surgical volumes between Asian and Western centers. Third, we focused on short-term morbidity; the long-term impact of NICT on quality of life and late-onset immune toxicities remains unknown. Fourth, pooling multiple anti-PD-1 agents into a single NICT group assumes class-equivalent surgical safety, an assumption not yet validated in comparative trials. Fifth, the dMMR/MSI-H stratum comprised only 22 patients, yielding unstable point estimates with minimal precision. Interaction tests were uniformly underpowered (all p > 0.05), precluding any inference regarding effect modification. These subgroup findings should be regarded as hypothesis-generating only. Finally, while pathological response is a strong surrogate for efficacy, we lacked data on PD-L1 and HER2 status in NCT group, and true validation of this strategy ultimately awaits mature overall survival data.

In conclusion, this propensity-matched analysis provides solid confirmatory evidence that the addition of immunotherapy to neoadjuvant chemotherapy for locally advanced GEJ adenocarcinoma maintains a favorable surgical safety profile. Rates of major complications were demonstrated statistically comparable to the control cohort, acknowledging that our sample size precludes ruling out rare but clinically meaningful harms. Coupled with a significant improvement in major pathological tumor regression, NICT demonstrates a favorable short-term clinical profile, balancing enhanced tumor downstaging with established surgical safety. While prospective randomized trials remain necessary to definitively establish optimal treatment paradigms, these data support the safety and feasibility of this approach. As the field moves forward, future efforts should prioritize identifying biomarkers to predict which patients will achieve profound pathological responses to NICT.

## Data Availability

The original contributions presented in the study are included in the article/**Supplementary Material**. Further inquiries can be directed to the corresponding author/s.
