# Supplementary material for: Does adding immunotherapy to neoadjuvant chemotherapy increase postoperative morbidity in gastroesophageal junction adenocarcinoma? A propensity score-matched study
Source: Front Immunol. 2026 Jul 3;17:1806803. doi: 10.3389/fimmu.2026.1806803 (PMC13375725; doi:10.3389/fimmu.2026.1806803)
Supplement: Supplementary file 1 [file Supplementaryfile1.docx]

### **Supplementary Table 1. Detailed Neoadjuvant Treatment Regimens in the Propensity Score-Matched Cohort (N=240)**

| ****Treatment Regimen**** | ****NICT (n=120)**** | ****NCT (n=120)**** |
| --- | --- | --- |
| ****Immunotherapy Agent**** |  |  |
| Pembrolizumab | 32 (26.7%) | -- |
| Sintilimab | 38 (31.7%) | -- |
| Tislelizumab | 28 (23.3%) | -- |
| Penpulimab | 22 (18.3%) | -- |
| ****Chemotherapy Backbone**** |  |  |
| Within NICT Cohort |  |  |
| CAPOX | 68 (56.7%) | -- |
| FOLFOX | 52 (43.3%) | -- |
| Within NCT Cohort |  |  |
| FLOT | 96 (80.0%) | -- |
| CROSS-like (carboplatin/paclitaxel) | 24 (20.0%) | -- |
| ****Concurrent Radiotherapy Details**** |  |  |
| Received Radiotherapy | 25 (20.8%) | 24 (20.0%) |
| ****Combined Regimen Detail**** |  |  |
| Pembrolizumab + CAPOX | 18 (15.0%) | -- |
| Pembrolizumab + FOLFOX | 14 (11.7%) | -- |
| Sintilimab + CAPOX | 22 (18.3%) | -- |
| Sintilimab + FOLFOX | 16 (13.3%) | -- |
| Tislelizumab + CAPOX | 16 (13.3%) | -- |
| Tislelizumab + FOLFOX | 12 (10.0%) | -- |
| Penpulimab + CAPOX | 12 (10.0%) | -- |
| Penpulimab + FOLFOX | 10 (8.3%) | -- |
| FLOT | -- | 96 (80.0%) |
| CROSS-like + Radiotherapy | -- | 24 (20.0%) |

****Abbreviations:**** NICT, Neoadjuvant Immunotherapy plus Chemotherapy; NCT, Neoadjuvant Chemotherapy; CAPOX, capecitabine and oxaliplatin; FOLFOX, infusional 5-fluorouracil, leucovorin, and oxaliplatin; FLOT, 5-fluorouracil, leucovorin, oxaliplatin, and docetaxel; CROSS-like, carboplatin and paclitaxel; IQR, Interquartile Range; RT, Radiotherapy.
